# The Effect of Hispidulin, a Flavonoid from *Salvia plebeia*, on Human Nasopharyngeal Carcinoma CNE-2Z Cell Proliferation, Migration, Invasion, and Apoptosis

**DOI:** 10.3390/molecules26061604

**Published:** 2021-03-14

**Authors:** Yiqun Dai, Xiaolong Sun, Bohan Li, Hui Ma, Pingping Wu, Yingping Zhang, Meilin Zhu, Hong-Mei Li, Minjian Qin, Cheng-Zhu Wu

**Affiliations:** 1 School of Pharmacy, Bengbu Medical College, 2600 Donghai Road, Bengbu 233030, China; daiyiqun25@126.com (Y.D.); sunxiaolong96@foxmail.com (X.S.); libohan1228@foxmail.com (B.L.); mahui9513@foxmail.com (H.M.); w18226552473@163.com (P.W.); zhangyingping1217@126.com (Y.Z.); zlyk521@126.com (M.Z.); athongmei@foxmail.com (H.-M.L.); 2 Department of Resources Science of Traditional Chinese Medicines, School of Traditional Chinese Pharmacy and State Key Laboratory of Natural Medicines, China Pharmaceutical University, Nanjing 210009, China

**Keywords:** hispidulin, nasopharyngeal carcinoma, apoptosis, invasion, migration

## Abstract

Nasopharyngeal carcinoma (NPC) is a common malignant head and neck tumor. Drug resistance and distant metastasis are the predominant cause of treatment failure in NPC patients. Hispidulin is a flavonoid extracted from the bioassay-guided separation of the EtOH extract of *Salvia plebeia* with strong anti-proliferative activity in nasopharyngeal carcinoma cells (CNE-2Z). In this study, the effects of hispidulin on proliferation, invasion, migration, and apoptosis were investigated in CNE-2Z cells. The [3-(4,5-dimethylthiazol-2-yl)-2,5-diphenyltetrazolium bromide] (MTT) assay and the colony formation assay revealed that hispidulin could inhibit CNE-2Z cell proliferation. Hispidulin (25, 50, 100 μM) also induced apoptosis in a dose-dependent manner in CNE-2Z cells. The expression of Akt was reduced, and the expression of the ratio of Bax/Bcl-2 was increased. In addition, scratch wound and transwell assays proved that hispidulin (6.25, 12.5, 25 μM) could inhibited the migration and invasion in CNE-2Z cells. The expressions of HIF-1α, MMP-9, and MMP-2 were decreased, while the MMPs inhibitor TIMP1 was enhanced by hispidulin. Moreover, hispidulin exhibited potent suppression tumor growth and low toxicity in CNE-2Z cancer-bearing mice at a dosage of 20 mg/kg/day. Thus, hispidulin appears to be a potentially effective agent for NPC treatment.

## 1. Introduction

Nasopharyngeal carcinoma (NPC) is one of the most common head and neck malignant tumor. It has a very unique distribution pattern and is much more prevalent in southeast Asia [1,2]. The conventional treatment for patients with NPC is chemotherapy and radiotherapy. Although the treatments have revealed good efficacy, high recurrence and metastasis become the major cause of treatment failure and have a poor prognosis [3]. Therefore, it is necessary to look for more effective therapeutic drugs for treating NPC. 

Traditional Chinese herbal medicine has become an important source of antitumor drugs and tumor adjuvant therapy with unique biological activities and few side effects. *Salvia plebeia* R. Brown is a traditional Chinese folk herb that belongs to the family Lamiaceae and is abundant in many countries, especially in China and India. It has been used as a folk herbal medicine for treating inflammatory diseases such as nephritis, pharyngitis, bronchitis, colds, and coughs, etc. [4,5,6]. In addition, there are also reports suggesting that *S. plebeia* has antitumor activity [7,8]. Although clinical treatment of tumors and the antitumor poperties of *S. plebeia* extracts have been reported, no study has reported the bioassay-guided isolation of the active principle responsible for the antitumor activity.

Flavonoids are the main chemical constituents of *S. plebeia*, which has multiple pharmacological effects and low toxicities. Hispidulin (4′,5,7-trihydroxy-6-methoxyflavone) is a flavonoid obtained from *S. plebeia* and has several pharmacological activities such as antioxidative, anti-inflammatory, anti-epileptic, hepatoprotective activities [9,10,11,12]. Noteworthy, accumulating studies have demonstrated the anti-cancer activity of hispidulin in renal cell carcinoma, gallbladder cancer, acute myeloid leukemia, hepatocellular carcinoma, and colorectal cancer by causing growth inhibition, cell cycle arrest, apoptotic activation, and metastasis suppression [13]. However, its anti-cancer activity on NPC remain poorly understood. Therefore, our study aimed to explore whether hispidulin is the main effective anti-cancer component in *S. plebeia* and investigated the anti-cancer effects of hispidulin on NPC.

## 2. Results

### 2.1. Isolation of Hispidulin from the Bioassay-Guided Fractionation of the EtOH Extract of S. plebeia

**Figure 1:**
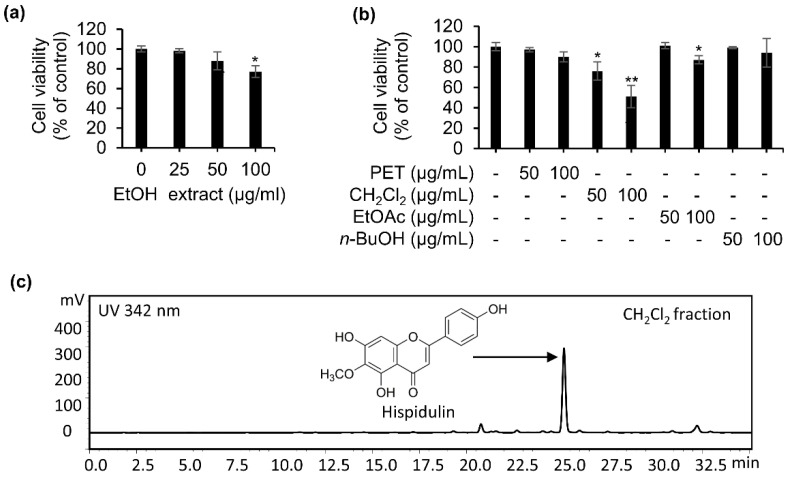
Effects of EtOH extract and fractions from *S. plebeia* on cell viability in CNE-2Z cells. (**a**) CNE-2Z cells were incubated for 24 h with various concentrations of EtOH extract of *S. plebeia*. (**b**) CNE-2Z cells were incubated for 24 h with various fractions from EtOH extract of *S. plebeia*. (**c**) High-performance liquid chromatography (HPLC) analysis of CH_2_Cl_2_ fraction from *S. plebeia* detected at 342 nm. * *p* < 0.05, ** *p* < 0.01 vs. control.

Firstly, we measured the cytotoxic effect of EtOH extract of *S. plebeia* on CNE-2Z cells. The crude extract significantly decreased CNE-2Z cell viability (Figure 1a). To look for what components are related to the anti-cancer effects of crude extract of *S. plebeia*, the crude extract was suspended in water and sequentially partitioned with petroleum ether, CH_2_Cl_2_, ethyl acetate, and n-butanol, respectively. The fractions were subjected to a cell proliferation assay. The results showed that CH_2_Cl_2_ fraction has stronger growth inhibition on CNE-2Z cells than other fractions (Figure 1b). Based on this result, CH_2_Cl_2_ fraction was chosen for further purification to find the active compound. High-performance liquid chromatography (HPLC) analysis revealed a major peak in the CH_2_Cl_2_ fraction (Figure 1c). The CH_2_Cl_2_ fraction was chromatographed on a silica gel column and Sephadex LH-20, and the major compound was gained. The structure of the compound was determined as hispidulin (Figure 1c) by comparing the nuclear magnetic resonance (NMR) data with previous literature [14]. The purity of hispidulin was higher than 98% by HPLC analysis.

### 2.2. Hispidulin Inhibits the Proliferation and Clonogenic Ability of CNE-2Z Cells

**Figure 2:**
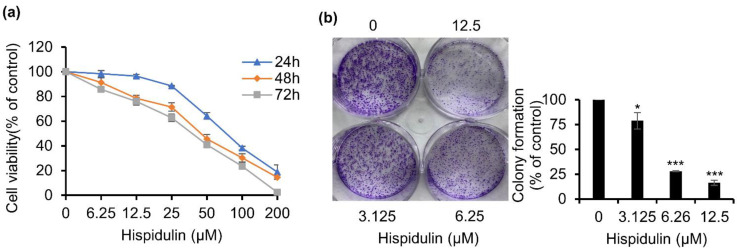
Anti-proliferative activities of hispidulin in CNE-2Z cells. (**a**) Effects of hispidulin on CNE-2Z cell viability for 24, 48, and 72 h by MTT assays. (**b**) Effects of hispidulin on the proliferative ability of CNE-2Z cells by colony-formation for 7 days. * *p* < 0.05, *** *p* < 0.001 vs. control.

The [3-(4,5-dimethylthiazol-2-yl)-2,5-diphenyltetrazolium bromide] (MTT) assay was applied to explore the potential effect of hispidulin on cell proliferation. CNE-2Z cells were incubated with different concentrations of hispidulin (0–200 μM) for 24, 48, and 72 h, then the number of living cells was determined. Hispidulin confirmed significantly inhibited CNE-2Z cell growth in a concentration and time-dependent manner (Figure 2a). Moreover, in a colony formation assay, we also found that hispidulin significantly decreased the colony formation ability in a concentration-dependent manner in CNE-2Z cells (Figure 2b). These results indicated that hispidulin significantly inhibits cell proliferation and survival of CNE-2Z cells.

### 2.3. Hispidulin Promotes the Apoptosis of CNE-2Z Cells

**Figure 3:**
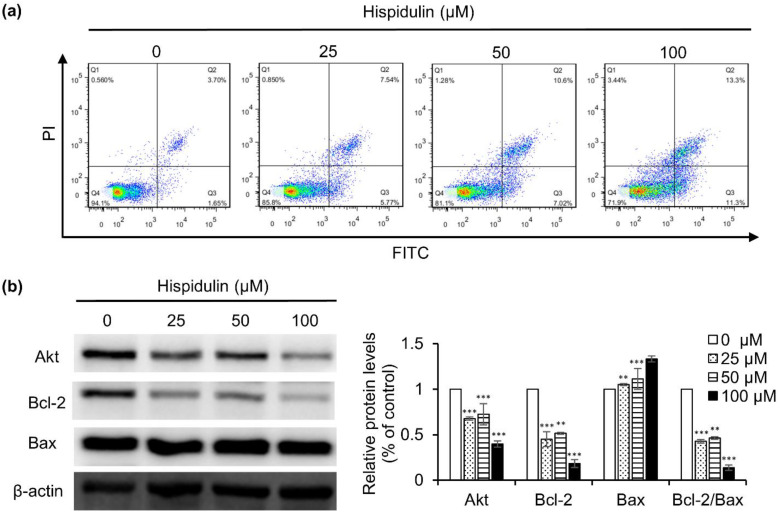
Hispidulin induced apoptosis in CNE-2Z cells. (**a**) CNE-2Z cells were cultured with hispidulin for 48 h and stained by Annexin V and PI staining, then apoptosis rate was determined by flow cytometry. (**b**) The expressions of Akt, Bcl-2, and Bax were analyzed by Western blot. ** *p* < 0.01, *** *p* < 0.001 vs. control.

In order to evaluate whether the inhibition over cell viability induced by hispidulin was related to the activation of the apoptotic pathway, we determined the number of apoptotic cells in different concentrations of hispidulin. Flow cytometry results showed that the rate of apoptosis induced by treating with various concentrations of hispidulin (25, 50, and 100 μM) was 13.3%, 17.1%, and 24.6%, respectively, while that of the control cells was 5.3% (Figure 3a). The data indicated that hispidulin induced apoptosis in a dose-dependent manner. Furthermore, we examined the effect of hispidulin on apoptosis-related proteins. The results suggested that hispidulin down-regulated the expression of Akt and up-regulated the ratio of Bax/Bcl-2 in a dose-dependent manner in CNE-2Z cells (Figure 3b).

### 2.4. Hispidulin Inhibits CNE-2Z Cells Migration and Invasion

**Figure 4:**
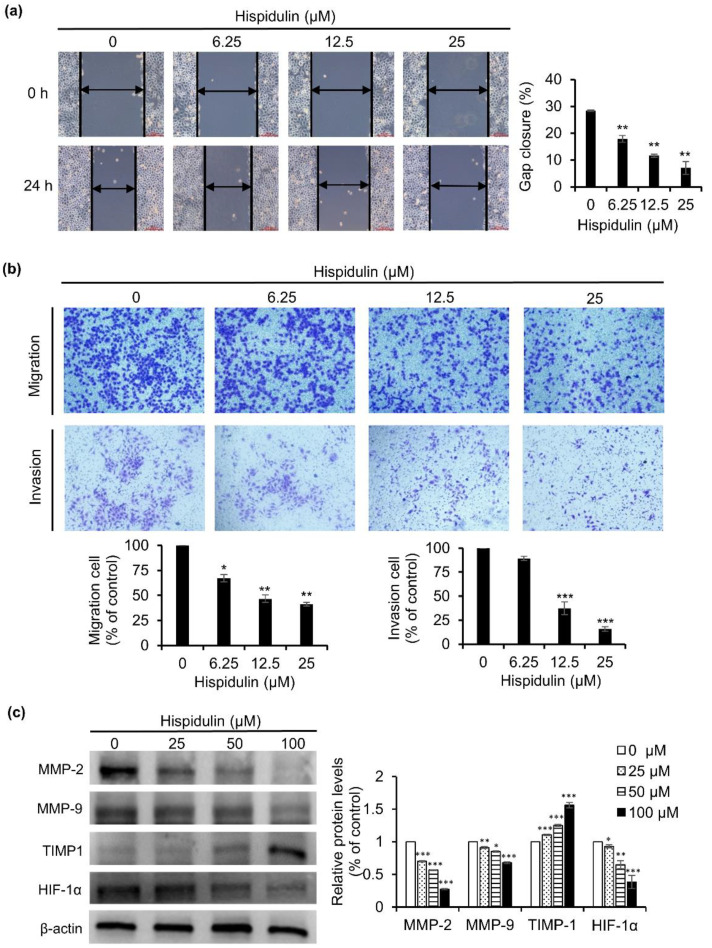
Hispidulin suppressed CNE-2Z cell migration and invasion. CNE-2Z cells were treated with various concentration of hispidulin for the indicated times. (**a**,**b**) Scratch wound and transwell invasion assays were performed to detect the migration and invasive abilities of CNE-2Z cells. (**c**) Western blot detected the expression of HIF-1α, MMP-2, MMP-9, and TIMP1. * *p* < 0.05, ** *p* < 0.01, *** *p* < 0.001 vs. control.

Next, the impact of hispidulin on CNE-2Z cell migration and invasion was tested by scratch wound assay and transwell chamber assay. CNE-2Z cells were treated with various concentrations of hispidulin for 24 h. As presented in Figure 4a, the results of the scratch wound assay revealed that the wound area was reduced in CNE-2Z cells treated with hispidulin compared to the control. In addition, a transwell assay was also used to test the effect of hispidulin on the migration of CNE-2Z cells. After treating with different concentrations of hispidulin, the migration rates were 67.3% (6.25 μM), 46.7% (12.5 μM), and 41.3% (25 μM) in comparison with the control group (Figure 4b). These results show that hispidulin inhibits the migration of CNE-2Z cells in a dose-dependent manner. A transwell assay was also used to estimate the effects of hispidulin on the invasion of CNE-2Z cells. The invasion rates were 89.3% (6.25 μM), 37.4% (12.5 μM), and 15.8% (25 μM), compared with the control cells (Figure 4b). The finding revealed that hispidulin inhibits the invasion of CNE-2Z cells in a dose-dependent manner. Correspondingly, after exposure to hispidulin for 48 h, biomarkers of cell invasion and migration, such as HIF-1α, MMP-2, and MMP-9, were down-regulated and MMPs inhibitor TIMP1 was up-regulated (Figure 4c). These results demonstrate that hispidulin can effectively inhibit the migration and invasion of CNE-2Z cells.

### 2.5. Hispidulin Suppresses Tumour Growth in Xenografted Nude Mice Model

**Figure 5:**
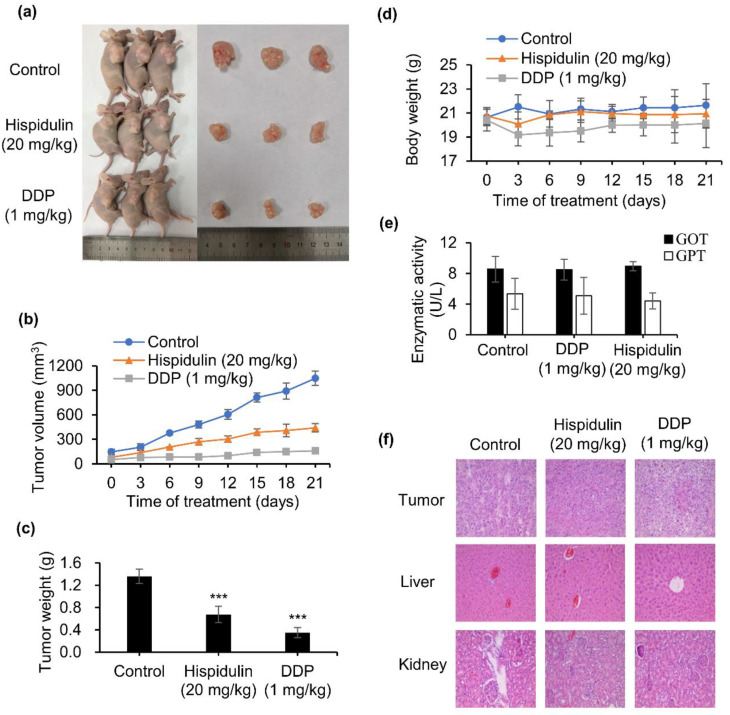
Hispidulin suppressed tumor growth in a xenograft model with CNE-2Z cells. (**a**) Representative images from each treatment group at the end of the experiment. (**b**) Tumor volumes of the mice were estimated. (**c**) The mice were sacrificed and the tumors were weighed. (**d**) Body weight of the mice was measured. (**e**) In vivo hepatotoxicity evaluation of hispidulin by glutamate oxaloacetate transaminase (GOT) and glutamate pyruvate transaminase (GPT) levels of blood serum samples, determined by assay kit. The GPT and GOT activities are expressed as U/L. (**f**) Hematoxylin and eosin (H & E) staining (original magnification 200×) of the tumor, liver, and kidney from the mice after treatment. *** *p* < 0.001 vs. control.

To further study whether hispidulin shows an antitumor effect in vivo in NPC, CNE-2Z tumor xenograft experiments were performed. The results showed that hispidulin at a dosage of 20 mg/kg/day could exert significant inhibition on tumor growth compared with the vehicle group (Figure 5a). As shown in Figure 5b, the tumor volume of hispidulin administration significantly reduced the tumor volume to 441.4 mm^3^, compared with 1048.1 mm^3^ in the control, dropping by 57.9%. Consistent with tumor volume data, hispidulin-treated groups indicated an obvious decrease in tumor weight (Figure 5c). In addition, our results showed that hispidulin did not affect body weight compared to the vehicle group (Figure 5d). Glutamate oxaloacetate transaminase (GOT) and glutamate pyruvate transaminase (GPT) are commonly used as biomarkers for the evaluation of hepatotoxicity [15]. Thus, we examined the levels of GOT and GPT using an activity assay, and the results showed that hispidulin had a negligible effect on GOT and GPT compared with the control group (Figure 5e). Moreover, hematoxylin and eosin (H & E) staining demonstrated that there was no obvious damage to the liver and kidney treatment with hispidulin at 20 mg/kg/day (Figure 5f). Altogether, the studies provided evidence that hispidulin can significantly inhibit tumor growth with low toxicity in vivo.

## 3. Discussion

In recent decades, the medicinal value of plants has increased. A large proportion of modern drugs are derived from medicinal plants. *S. plebeia*, a species of genus Salvia, has been used as a traditional folk medicine in treating inflammatory diseases and cancer, and its main substances are flavonoids [16]. In this study, hispidulin was obtained from *S. plebeia* by a bioassay-guided fractionation approach. Research evidence demonstrates that hispidulin has pleiotropic biological activities, such as antioxidant, anti-inflammatory, anti-osteoporotic, anti-thrombotic, and neuroprotective activities [17,18,19,20,21]. In addition, many studies have shown that hispidulin exerts anti-tumor effects in many kinds of cancers in vivo and in vitro. However, whether hispidulin has the ability to inhibit tumor growth and tumor metastasis in NPC is still unclear. The results of the present study for the first time revealed that hispidulin induced apoptosis inhibited the proliferation and metastasis on CNE-2Z cells. 

MTT experiments showed that hispidulin could significantly reduce the survival rate of CNE-2Z cells. The results showed that hispidulin inhibited the growth of CNE-2Z cells potently in vitro. Similarly, the anti-NPC effect of hispidulin in vivo was consistent with the efficacy in vitro. In xenograft tumor mice, the oral administration of 20 mg/kg/day of hispidulin resulted in a 50.1% reduction in tumor weight, without toxic effects, on mouse body weight, exhibiting its potential as an effective candidate agent in the treatment of NPC.

Apoptosis is crucial for cell proliferation and tumor growth [22]. In this study, flow cytometry results showed that hispidulin could trigger apoptotic response on CNE-2Z cells after 48 h of treatment. In addition, the apoptotic proteins, Bcl-2 (antiapoptotic) and Bax (proapoptotic) are important apoptosis indexes. The results of the present study showed that hispidulin can up-regulate the ratio of Bax/Bcl-2. This is in line with several previous studies on hispidulin-induced apoptosis in SMMC7721 cells, PANC-1 cells, and HT29 cells [23,24,25]. It has been shown that the phosphatidylinositol-3 OH kinase (PI3K)/Akt pathway is one of the critical signaling pathways, which contributes to cancer survival, apoptosis, and metastasis. In particular, Akt is a serine/threonine protein kinase; it plays a critical role in control the balance between cell survival and apoptosis [26]. Activated Akt up-regulate Bax as well as modulate Bcl-2 protein to trigger apoptosis [27]. The results of the present study showed that hispidulin down-regulated Akt expression and up-regulated the ratio of Bax/Bcl-2, suggesting that hispidulin stimulates CNE-2Z cell apoptosis, possibly through modulation of the Akt signaling pathway.

The PI3k/Akt signaling pathway also plays a key role in cell migration and invasion through affecting multiple downstream targets. One of the downstream protein kinases, mTOR (mammalian target of rapamycin), can active the hypoxia-inducible factor 1α (HIF-1α), thereby regulating the expression of matrix metalloproteinase (MMP)-9 and the tissue inhibitor of matrix metalloproteinase (TIMP)-1 to prompt invasion and migration [28,29,30]. The present data demonstrated that hispidulin reduced the expression of HIF-1α, MMP-9, and MMP-2 and increased the expression of TIMP1, possibly through suppressing the PI3K/AKT/HIF-1α pathways, thereby inhibiting the migration and invasion of CNE-2Z cells.

In conclusion, hispidulin was obtained from *S. plebeia* by a bioassay-guided approach. We found, for the first time, that hispidulin exerts a potent anti-cancer effect in human nasopharyngeal carcinoma CNE-2Z cells. Hispidulin can promote apoptosis and inhibit migration and invasion of CNE-2Z cells in vitro and inhibit tumor growth in vivo. Furthermore, hispidulin might induce apoptosis and inhibited invasion and migration in CNE-2Z cells by regulating the PI3K/AKT pathways. We believe that hispidulin may be a potential agent for the treatment of NPC.

## 4. Materials and Methods

### 4.1. Reagents

Roswell Park Memorial Institute (RPMI)-1640 was purchased from Hyclone (Logan, UT, USA). Fetal bovine serum (FBS) was purchased from Sijiqing (Hangzhou, China). 3-(4,5-Dimethylthiazol-2-yl)-2,5-diphenyl tetrazolium bromide (MTT) and dimethyl sulfoxide (DMSO) were purchased from Sigma–Aldrich (St. Louis, MO, USA). An annexin V/PI staining kit was purchased from BestBio Tech. Co., Shanghai, China. The specific primary antibodies against Akt, Bax, Bcl-2, and β-actin were purchased from Proteintech (Rosemont, IL, USA) and antibodies against HIF-1α, TIMP1, MMP-2, and MMP-9 were purchased from Abcam (Cambridge, MA, USA). The secondary antibodies used in this study were goat anti-rabbit IgG-HRP or anti-mouse IgGHRP (BioSharp, Hefei, China).

### 4.2. Plant Material and Extraction

The basal leaves of *S. plebeia* R. Br. were collected from Bengbu City, Anhui Province, the People’s Republic of China, in February 2018 and identified by Professor Minjian Qin (Department of Resources Science of Traditional Chinese Medicines, China Pharmaceutical University, Nanjing, China). The voucher specimen (No. SP 2018002) was deposited in the Natural Medicinal Chemistry Laboratory of Bengbu Medical College, China. The air-dried basal leaves of *S. plebeia* (5 kg) were powdered and percolated with 95% ethanol (20 L) at room temperature. The solvent was evaporated under reduced pressure to yield a residue (310 g). The crude extract was suspended in water and sequentially partitioned with petroleum ether, CH_2_Cl_2_, ethyl acetate, and n-butanol, respectively. The filtration of the extracted solutions and evaporation under reduced pressure gave a petroleum ether extract (24.1 g), a CH_2_Cl_2_ extract (10.3 g), an ethyl acetate extract (42.8 g), and an *n*-butanol extract (39.6 g).

### 4.3. Cell Line and Cell Culture

The human nasopharyngeal carcinoma cell line CNE-2Z was purchased from the Shanghai Cell Bank (Shanghai, China) and cultured in RPMI-1640 medium supplemented with 10% fetal bovine serum and antibiotics (1% penicillin/streptomycin) and incubated at 37 °C in a 5% CO_2_ humidified atmosphere.

### 4.4. Cell Viability Assay 

The cell viability was determined by using MTT assay. The CNE-2Z cells were seeded at a density of 6000 cells per well in 96-well plates and treated with various concentrations of hispidulin for 24, 48, and 72 h. At the end of each time point, a 10 µL aliquot of MTT was added and incubated for 4 h. A volume of 100 µL DMSO for 10 min was used to dissolve the formazan crystals. The absorbance (A) was determined using a microplate spectrophotometer at 570 nm (Bio-Rad, Hercules, CA, USA). Cell viability was described as the relative percentage of the control.

### 4.5. Colony Formation Assay 

The CNE-2Z cells were seeded in 6-well culture plates at 4000 cells/well and allowed to attach overnight, then exposed to various concentrations of hispidulin (3.125, 6.25, 12.5 μM) under standard cell culture conditions for 7 days. After treatment, washing twice with phosphate buffer saline (PBS), the colonies were fixed with paraformaldehyde for 15 min, stained with crystal violet for 10 min, washed with double-distilled water, dried at room temperature, and then photographed.

### 4.6. Flow Cytometry Analysis of Apoptosis 

Cell apoptosis was performed using an Annexin V/PI staining kit. CNE-2Z cells were cultured in 6-well plates at a density of 3 × 10^5^ cells/well overnight, then various concentrations of hispidulin (25, 50, and 100 µM) were added to the cells. After 48 h, cells were harvested at a density of 5 × 10^5^ cells/mL and incubated with Annexin V-FITC and propidium (PI) at room temperature in the dark for 15 min before detection using a flow cytometry (BD Biosciences, Franklin Lakes, NJ, USA).

### 4.7. Western Blotting Analysis

CNE-2Z cells were treated with different concentrations of hispidulin (25, 50, and 100 μM) for 48 h. After treatment, cells were harvested and lysed in radio immunoprecipitation assay (RIPA) buffer for 30 min on ice. Following 12,000 rpm at 4 °C for 15 min, the supernatants were harvested for protein quantification using a bicinchoninic acid assay kit. Then, equal amounts of total protein were separated using sodium dodecyl sulfate polyacrylamide gel electrophoresis (SDS-PAGE) and transferred to polyvinylidene difluoride (PVDF) membranes and blocked with 5% skimmed milk for 2 h at room temperature. Then, the PVDF membranes were immunoblotted with specific antibody (Akt, Bax, Bcl-2, HIF-1α, MMP-2, MMP-9, TIMP1, or β-actin) overnight at 4 °C, followed by incubating with the corresponding goat anti-rabbit or anti-mouse antibodies at room temperature for 2 h. Protein levels were measured by an enhanced chemiluminescence kit (Millipore, Burlington, MA, USA) and visualized by gel imaging equipment (Bio-Rad, Hercules, CA, USA).

### 4.8. Scratch Wound, Transwell Migration, and Invasion Assays

The CNE-2Z cells were seeded in 6-well culture plates at 6 × 10^5^ cells/well. The wound was generated using 200 μL pipette tip in each well of six-well plates on 90% confluence. After washing, cells were treated with different concentrations of hispidulin (6.25, 12.5, and 25 μM) for 24 h, and then observed and photographed with an inverted microscope. The healing percentages was measured with Image J software (National Institutes of Health, Bethesda, MD, USA).

Transwell migration and invasion assays were performed using transwell 24-well plates with 8 µm pore membrane inserts (Corning, Corning, NY, USA). For invasion assays, the transwell chambers were pre-coated with 50 μL of matrigel (BD, Franklin Lakes, NJ, USA). The CNE-2Z cells (1 × 10^5^) in 200 μL of serum-free medium containing different concentrations of hispidulin (6.25, 12.5 and 25 μM) were placed in the upper chamber, and 800 μL of RPMI-1640 medium supplemented by 15% FBS was placed in the lower chamber. After 24 h incubation, the cells were fixed in 4% paraformaldehyde for 15 min and stained with 0.1% crystal violet for 15 min. Then non-invasive cells on the upper chamber were removed with a cotton swab. Finally, the invaded cells were enumerated under a light microscope (200×). For migration assays, the cells were carried out as mentioned above, except that the upper chamber was without matrigel.

### 4.9. Xenograft Model

BALB/c-nu mice (5–6 weeks old) were purchased from Shanghai SLAC Laboratory Animal Co., Ltd. (Shanghai, China). The animal care and animal experiments were performed in accordance with and approved (permission number (2019) 014) by the Animal Management and Ethics Committee of Bengbu Medical College. CNE-2Z cells (4 × 10^6^) suspended in 0.2 mL PBS were injected subcutaneously into the right flank of each mouse. Growth of the tumor was measured with calipers twice a week. Tumor volume was calculated with the formula:

volume (mm^3^) = length × width^2^/2

When the tumors reached about 80 mm^3^, the tumor-bearing mice were randomly divided into 3 groups (*n* = 3/group): vehicle control, hispidulin (20 mg/kg), and DDP (1 mg/kg). Drugs were injected intraperitoneally every day. Meanwhile, the body weight of the mice was recorded during the course. After 21 days of treatment, the animals were sacrificed and the primary tumor, liver, and kidney were removed from each group of mice and stained with hematoxylin and eosin (H & E). In addition, blood samples were collected for determination of glutamate oxaloacetate transaminase (GOT) and glutamate pyruvate transaminase (GPT) levers in the serum. Cisplatin (DDP) was administrated as a positive control drug.

### 4.10. Statistical Analysis

The Student’s *t*-test using SPSS 16.0 software (SPSS Inc., Chicago, IL, USA) was used for analysis. *p* < 0.05 denotes a significant difference. Data were expressed as the mean ± standard deviation. All the samples were measured at least in triplicate.

## Data Availability

The data presented in this study are available on request from the corresponding authors.

## Sample Availability

Samples of the compounds are available from the authors.
